# A review of constraints and adjustable parameters in microgrids for cost and carbon dioxide emission reduction

**DOI:** 10.1016/j.heliyon.2024.e27489

**Published:** 2024-03-06

**Authors:** Mohammed Amine Hoummadi, Hala Alami Aroussi, Badre Bossoufi, Mohammed Karim, Saleh Mobayen, Anton Zhilenkov, Thamer A. H. Alghamdi

**Affiliations:** a LIMAS Laboratory, Faculty of Sciences Dhar El Mahraz, Sidi Mohammed Ben Abdellah University, Fez, 30003, Morocco; b LGEM Laboratory, Higher School of Technology, Mohamed First University, Oujda, 60000, Morocco; c Graduate School of Intelligent Data Science, National Yunlin University of Science and Technology, 123 University Road, Section 3, Douliou, Yunlin, 640301, Taiwan; d Department of Cyber-Physical Systems, St. Petersburg State Marine Technical University, 190121, Saint-Petersburg, Russia; e Wolfson Centre for Magnetics, School of Engineering, Cardiff University, Cardiff, CF24 3AA, UK; f Electrical Engineering Department, School of Engineering, Al-Baha University, Al-Baha, 65779, Saudi Arabia

**Keywords:** Energy costs, Energy demand, Load, Load forecast, Microgrid, Power rate

## Abstract

In a world grappling with escalating energy demand and pressing environmental concerns, microgrids have risen as a promising solution to bolster energy efficiency, alleviate costs, and mitigate carbon emissions. This article delves into the dynamic realm of microgrids, emphasizing their indispensable role in addressing today's energy needs while navigating the hazards of pollution. Microgrid operations are intricately shaped by a web of constraints, categorized into two essential domains: those inherent to the microgrid itself and those dictated by the external environment. These constraints, stemming from component limitations, environmental factors, and grid connections, exert substantial influence over the microgrid's operational capabilities. Of particular significance is the three-tiered control framework, encompassing primary, secondary, and energy management controls. This framework guarantees the microgrid's optimal function, regulating power quality, frequency, and voltage within predefined parameters. Central to these operations is the energy management control, the third tier, which warrants in-depth exploration. This facet unveils the art of fine-tuning parameters within the microgrid's components, seamlessly connecting them with their surroundings to streamline energy flow and safeguard uninterrupted operation. In essence, this article scrutinizes the intricate interplay between microgrid constraints and energy management parameters, illuminating how the nuanced adjustment of these parameters is instrumental in achieving the dual objectives of cost reduction and Carbon Dioxide emission minimization, thereby shaping a more sustainable and eco-conscious energy landscape. This study investigates microgrid dynamics, focusing on the nuanced interplay between constraints and energy management for cost reduction and Carbon Dioxide minimization. We employ a three-tiered control framework—primary, secondary, and energy management controls—to regulate microgrid function, exploring fine-tuned parameter adjustments for optimal performance.

## Nomenclature

*E*: The efficiency in %
*MG*: microgrid
*CO2*: Carbon Dioxide
*SOC*: State Of Charge
*DOD*: Depth Of Discharge
*AI*: Artificial Intelligence
*IOT*: Internet Of Things
*CDER*: Converter-Interfaced Distributed Energy Resources
*DR*: Demand Response

## 1 Introduction

The new century has ushered in a transformative era marked by an exponential surge in global energy demand, driven by urbanization, industrialization, and technological advancements [1]. This escalating demand has, however, given rise to a formidable environmental challenge the alarming escalation of pollution and carbon emissions [[2], [3], [4]]. In response to this, microgrids have emerged as a pivotal force in the global energy landscape, not only as technological innovations but as a paradigm shift in energy perception and utilization [5,6,7]. This first section aims to provide a broad overview of the challenges posed by increased energy demand, the environmental consequences, and the role of microgrids in addressing these challenges.

**Fig. 1:**
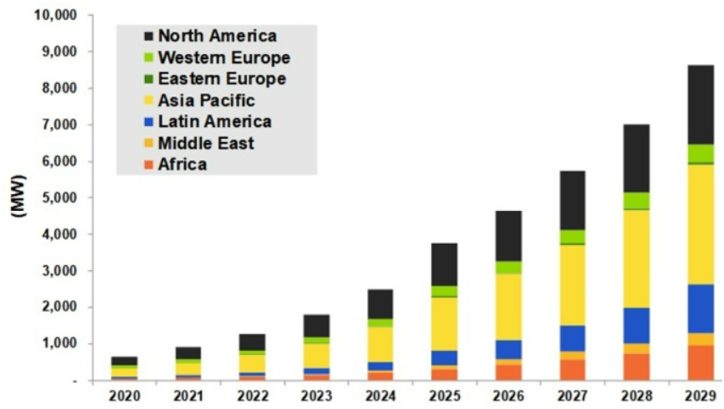
Global Microgrid adoption and decarbonization statistics.

The second part delves into the varied methods and measures adopted by countries worldwide in addressing the challenges of microgrid decarbonization. This involves a comprehensive exploration of global initiatives, policies, and statistics related to the deployment and impact of microgrids. Graphical representation, such as Fig. 1, illustrates key statistics or a comparative analysis of microgrid adoption and decarbonization efforts across different regions. This section aims to provide a nuanced understanding of the diverse approaches taken by countries, shedding light on successful strategies and potential areas for improvement [8] (Fig. 1).

The third section situates the current paper within the context of existing literature, particularly focusing on papers addressing grid decarbonization. Relevant studies, such as [1,2], are critically reviewed to highlight gaps and areas that remain unexplored. The authors justify the significance of their work by articulating how the present paper brings novel insights, introduces topics not covered in previous literature, or approaches microgrid decarbonization from a distinctive angle. This section aims to position the current research within the evolving landscape of grid decarbonization studies [[9], [10], [11]]. This section of the paper delineates the principal contributions of our study, aiming to underscore its distinctive qualities and relevance within the domain of microgrid decarbonization. We have restructured the introduction to provide a comprehensive background on microgrids, cost considerations, carbon emissions, and the significance of reduction efforts. Specifically, we highlight the escalating imperatives for decarbonization, driven by the dual goals of mitigating environmental impact and bolstering economic efficiency. Within this context, our study introduces an innovative methodology or technology aimed at advancing microgrid decarbonization efforts. Additionally, we advocate for the integration of Artificial Intelligence (AI) and Internet of Things (IoT) technologies to bolster the adaptability and intelligence of microgrids. Furthermore, we delve into the exploration and optimization of critical parameters vital for sustainable energy management within microgrid systems, addressing the pressing need to balance energy generation, storage, and demand to minimize costs and carbon emissions. Through a comprehensive analysis of global approaches to microgrid decarbonization, we aim to highlight successful strategies while identifying potential areas for enhancement, thereby contributing to the advancement of knowledge in the field [20, 21,22]. Additionally, our study addresses lacunae in existing literature by tackling specific topics or aspects previously overlooked, such as the role of emerging technologies and the integration of renewable energy sources in microgrid operations. These delineated sections furnish a structured and informative introduction, laying a robust foundation for the subsequent discourse on microgrid decarbonization within the paper. Against the backdrop of escalating imperatives for decarbonization, our study heralds a novel contribution: a comprehensive portal designed to aggregate and disseminate the latest research articles focusing on parameters pivotal for concurrently curbing costs and CO2 emissions in microgrid systems. By elucidating the intricate nexus between fiscal considerations and ecological imperatives, our portal aspires to empower stakeholders with actionable insights, facilitating informed decision-making processes and fostering the adoption of sustainable practices conducive to the realization of global environmental goals.

## 2 Microgrid constraints

**Fig. 2:**
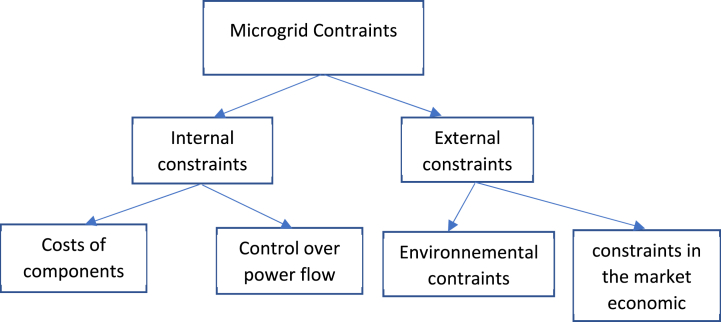
The various constraints of a microgrid.

**Fig. 3:**
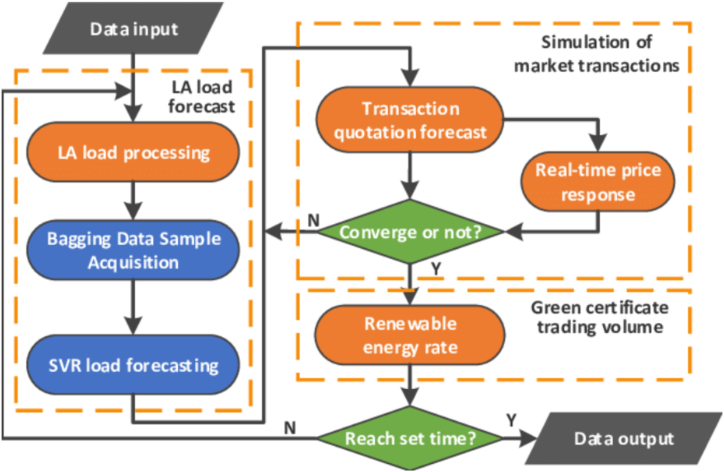
Chart control of power flow.

The constraints that affect a microgrid can be categorized into two main groups: those stemming from inherent limitations [24, 12] within the microgrid itself and those imposed by the external environment [13]. In the first category, microgrids face challenges such as high initial setup costs and the complex integration of diverse components and systems [13,14]. The second category involves external environmental constraints, including regulatory and policy frameworks that vary from region to region and can pose hurdles for obtaining necessary approvals and fostering microgrid development. Clarifying and simplifying these regulations [15] is crucial for facilitating the implementation and widespread adoption of microgrids, thereby mitigating these constraints [15,16]. Fig. 3 displays the hierarchical arrangement of these constraints (Fig. 2).

### 2.1 Costs of components

The cost C (C is the symbol of Cost in $/kw it's explained in nomenclature in the first page) of a component depends on different important parameters such as size, materials, market.

The cost of a component [26, 29, 17] can be expressed as follow (equation (1)):

(1) C=(Ci+Ic+Lt*Mc)/(Pn.Lt)

Where: *C is* The cost in $/kwh

*Ci* is Initial cost in $

*Mc* is the Maintenance cost in $/year.

*Ic* is the Installation cost in $

*Pn* is the Nominal power in KW.

*Lt* is the Lifetime of the component in years.

### 2.2 Control over power flow

The control over power flow in a microgrid involves various aspects, and demand forecasting and load balancing are integral components of this control mechanism. Demand forecasting refers to the prediction of future energy consumption based on historical data, patterns, and other relevant factors. Load balancing, on the other hand, involves distributing the electrical load evenly across the microgrid to ensure optimal utilization of resources and prevent overloading of specific components. Together, these elements contribute to effective power flow control within the microgrid. Demand forecasting [18] is the process of estimating future consumer demand for a product or service based on historical data and analysis. In the context of the energy industry, demand forecasting specifically refers to predicting the amount of electricity or energy that will be consumed in a given area over a defined period, usually hours, days, or even years in advance. This prediction is critical for utility companies, grid operators, and energy suppliers to efficiently plan and manage their resources, ensuring a reliable and cost-effective supply of electricity. Demand forecasting involves analyzing historical consumption patterns, weather data, economic factors, and other variables to create models that project future energy demand. Accurate demand forecasting helps avoid overloading the power grid, reduces energy waste [19,20], and ensures that the right amount of electricity is generated and distributed, ultimately leading to cost savings and improved energy efficiency [30, 31, 21]. Load balancing [22], in the context of energy distribution and microgrids, is the process of evenly distributing electrical loads or power consumption across various sources and distribution pathways within a network. This practice is essential to ensure the stable and efficient operation of the electrical grid, particularly when dealing with a mix of energy sources, demand fluctuations, and variable loads. Load balancing aims to match the supply of electricity with the demand in real-time [[22], [23], [24]], preventing power outages, overloads, or disruptions. It involves continuously adjusting the flow of electricity and the utilization of energy sources to maintain grid stability. For example, if a microgrid incorporates solar and wind power, load balancing may involve using energy storage systems or switching to a backup generator when renewable energy production is low [32, 33, 25]. It optimizes the utilization of resources and minimizes waste, ultimately enhancing grid reliability and sustainability [26,27].

While demand forecasting and load balancing are complex processes that involve the analysis of data and variables, they don't have specific equations. Instead, they rely on various mathematical and statistical models to make predictions and optimize load distribution. These models can include linear regression, time series analysis, machine learning algorithms, and optimization techniques [28].

For demand forecasting, a simple linear regression equation might look like this (equation (2)):

(2) Y=a+bX

Where:

*Y* the predicted energy demand in KWh.

*X* one or more relevant variables, such as time, weather conditions, and economic indicators

*a* The intercept

*b* the slope of the regression line.

Load balancing involves dynamic adjustments in real-time and often relies on control algorithms rather than a fixed equation. These algorithms consider the current load, available energy sources, and other factors to make decisions about load distribution. For example, a load balancing algorithm for a microgrid might decide to draw power from a battery during peak demand to avoid overloading the grid. The specific equations and algorithms used for demand forecasting and load balancing can be highly customized and depend on the complexity of the system, the available data, and the goals of the energy management process. These equations and algorithms are typically implemented using software and computational tools rather than simple mathematical expressions [[29], [30], [31]]. Fig. 3 shows how power flow can be controlled using data and software.

### 2.3 Environmental constraints

Environmental constraints affecting microgrids can encompass a range of challenges associated with sustainability and the eco-conscious operation of these localized power systems. One significant environmental constraint involves the reliance on renewable energy sources, such as solar and wind, which are inherently variable and dependent on weather conditions. This variability in energy generation can pose challenges in maintaining a consistent power supply and may necessitate the integration of energy storage systems to bridge gaps in production [32]. Additionally, the environmental impact of energy sources is a critical constraint [33,34]. The choice of energy generation methods, including the life cycle analysis of materials, emissions, and land use, plays a pivotal role in the overall sustainability of a microgrid. Balancing the economic viability of renewable energy sources with their environmental benefits can be complex. Furthermore, microgrids need to consider ecological factors, as their installation may impact local ecosystems and land use [33, 34, 35]. Sustainable practices, such as minimizing land disturbance and preserving biodiversity, are crucial in mitigating these constraints [[35], [36], [37]]. Addressing environmental constraints in microgrid development is essential for achieving the dual goals of sustainability and energy reliability. It involves the strategic integration of renewable energy sources, environmental impact assessments, and eco-conscious practices to ensure that microgrids contribute positively to both the local and global environment [38,39]. Take note that the following models are employed to characterize the variability of random variables such as wind speed and sun radiation. In the specified context of [renewable energy forecasting], these models consider various influencing factors, like meteorological parameters, geographical elements, and environmental conditions. The model for wind speed captures the interplay of these factors, while the sun radiation model incorporates considerations such as time of day and other environmental variables. These models provide a framework for understanding and predicting the fluctuations in wind speed and sun radiation, essential for purposes such as optimizing energy production.

#### 2.3.1 Wind equation

The mathematical law commonly used to model wind speed is the Weibull distribution. It describes the probability of wind speed occurring at a given velocity and is formulated as follows (equation (3)):

(3) $$f\left(v\right)=\left(c\;/\;k\right)\;*\;(v\;/\;k\hat{)}\left(c\;-\;1\right)\;*\;\exp\left(-\left((v\;/\;k\hat{)}c\right)\right)$$

where:

*f(v)* the probability density function of wind speed at velocity (v).

*c* the Weibull shape parameter, determining the shape of the distribution

*k* the Weibull scale parameter, influencing the scale of wind speeds.

This mathematical law is frequently used to model the distribution of wind speed in applications such as wind energy, weather forecasting, and the design of structures to withstand wind loads. It characterizes the variability of wind speeds in a specific region [40].

#### 2.3.2 Solar irradiance equation

The solar irradiance equation, which describes the solar radiation received at a particular location on Earth, is known as the solar insolation equation. It is often expressed as (equation (4)):

(4) I=Isc(DNI/DNIsc)

Where:

*I* the solar irradiance (solar radiation) at the location in watts per square meter (W/m^2^).

*Isc* the solar constant, which is approximately 1361 W/m^2^ and represents the solar radiation intensity outside Earth's atmosphere.

*DNI* the Direct Normal Irradiance, which is the solar radiation received on a surface perpendicular to the Sun's rays on a plane horizontal to the Earth's surface. It is typically measured in W/m^2^

*DNIsc* the solar constant on a surface perpendicular to the Sun's rays.

This equation allows you to calculate the solar irradiance at a specific location on Earth by considering the solar constant and the direct normal irradiance. The actual solar irradiance may vary depending on factors such as time of day, weather conditions, and the location's geographical coordinates [41].

### 2.4 Economic constraints in the market

Economic constraints in the microgrid market encompass a spectrum of considerations that impact the feasibility and sustainability of these decentralized energy systems. Among the most significant factors are economic variables like inflation and the price of combustible fuels. Inflation can erode the purchasing power of investments in microgrid infrastructure [[42], [43], [44]], potentially rendering them less economically viable over time. Moreover, the price of combustible fuels, which is closely linked to fluctuations in global energy markets, affects the operating costs of backup generators or other conventional energy sources within a microgrid. As these prices rise, it can escalate the overall cost of microgrid operation and undermine its cost-effectiveness [42,43]. Indirect costs also form a part of the economic constraints. These encompass expenses such as maintenance, repair, and equipment replacement, which can accumulate over the lifecycle of a microgrid. Unforeseen events, such as extreme weather conditions or equipment failures, can lead to sudden spikes in operational costs. Furthermore, market dynamics, including fluctuating electricity prices and regulatory changes, can affect the potential for microgrid owners to sell excess energy back to the grid or benefit from incentives [43,44]. Overall, economic constraints in the microgrid market necessitate thorough economic modeling, risk assessments, and proactive financial planning. Mitigating these constraints often involves diversified energy sources, efficient equipment maintenance, and strategies to manage energy costs effectively, such as demand-side management and revenue-generating opportunities, to ensure the long-term economic sustainability of microgrid projects [45,46]. The inflation rate can be calculated using the following equation (5):

(5) InflationRate=[(CPI−CPIold)/CPIold]*100

where “Consumer Price Index (CPI)_new" represents the CPI for a specific period, such as the current month or year, and “Consumer Price Index (CPI)_old" represents the CPI for a previous period, such as the previous month or year. The formula calculates the percentage change in the CPI between two time periods and expresses it as the inflation rate. The CPI measures the average change in prices paid by consumers for a basket of goods and services, making it a key indicator for tracking inflation [47].

### 2.5 Some limitations and constraints in the practice

Table 1 below provides a comprehensive list of constraints encountered by the microgrid along with a brief description of each constraint.

**Table 1:** Microgrid constraints in practice.

Constraint	Explanation	Reference
Power balance	Consumption and generation must coincide. There are some exceptions when it comes to chance-constrained issues. Occasionally, reactive power is included.	[2,5,6,[18], [19], [20], [21], [22], [23], [24], [25], [26], [27], [28], [29], [30], [31], [32], [33], [48], [49], [50], [51]]
Load demand	It is believed that the actual load exceeded the anticipated load.	[48,34]
Power rate	Maximum and lowest power recommendations from the manufacturer.	[ [2,5,6,[48], [49], [50],[18], [19], [20], [21], [22], [23], [24], [25], [26]], [[28], [29], [30], [31],^33^,^35^]]
Dynamic power rate	dynamic charging/discharging ratio, measured by the energy stored in the final stage or state of charge (SOC)	[51,20,30,31]
Apparent power	limitations on the apparent power of power flow, MG lines, or generators.	[19,[35], [36], [37]]
Unbalance	Maximum neutral current for converters connecting MG and CDERs	[2]
Voltage level	limitations on the power buses and/or terminal voltage of DERs	[2,6,19,36,[38], [39], [52]]
Power ramping	Maximum power ramping up/down suggestions from the manufacturer	[ [2,50,19,21,30,31], [33,36,[40], [41], [42], [43]]]
On/off min time	The shortest duration for which a CDER maintains its on/off state	[2,5,42,[44], [45], [46]]
Max. startups and shutdowns	Maximum quantity of CDER starting and shutdown events.	[[43], [44], [45]]
Power Reserve contraint	a power reserve in case of unexpected fluctuations in energy availability or load demand.	[5,47]
Diesel volume	an absolute minimum of diesel in the DG tank.	[48,25,53]
Excess of génération	Demand response (DR) systems, dump loads, and ESS will receive the extra generation.	[18,30,31,42]
Controllable load limits	restrictions on the energy (deferrable loads), power, or maximum percentage of interruption at each time step for controllable loads.	[46,51,54,56,65,68, 76,82]
Soc limits	Manufacturer guidelines on SOC limitations for batteries and fuel cells (FCs).	[2,7,46,48,51–59, 61–65,68]
Initial and/or final SOC	SOC minimum at start and finish of a term or maximum constraints on the daily number of operations and/or the maximum DOD in batteries	[7,51–53,56,57,61, 68,70,83]
Depth of discharge (DOD) and/or max. number of Daily opérations	Restrictions on the daily number of operations and/or the maximum DOD in batteries.	[29,43]
Pollution quantity	Limits on the highest amount of pollutants that can be released into the atmosphere. In grid-connected MGs, pollution from the main grid is occasionally taken into account as well.	[[54], [55], [56]]

Table 1 presents a comprehensive overview of the diverse constraints that microgrids encounter, each accompanied by a succinct description outlining its nature. These constraints form a crucial dimension in understanding the challenges and intricacies faced by microgrid systems. Power balance, a fundamental constraint, necessitates meticulous management to ensure that the generation capacity aligns seamlessly with load demand. Load demand, another key factor, reflects the varying needs for electricity within the microgrid, requiring dynamic adaptation for optimal performance. The table further delves into constraints related to environmental factors, grid dynamics, and technical limitations, shedding light on the multifaceted nature of challenges that microgrids navigate. This comprehensive listing provides a valuable resource for researchers, practitioners, and policymakers to gain insights into the intricate web of constraints influencing microgrid functionality, ultimately contributing to the development of strategies for more resilient and efficient energy systems.

### 2.6 Constraints and optimization of the microgrid

**Fig. 4:**
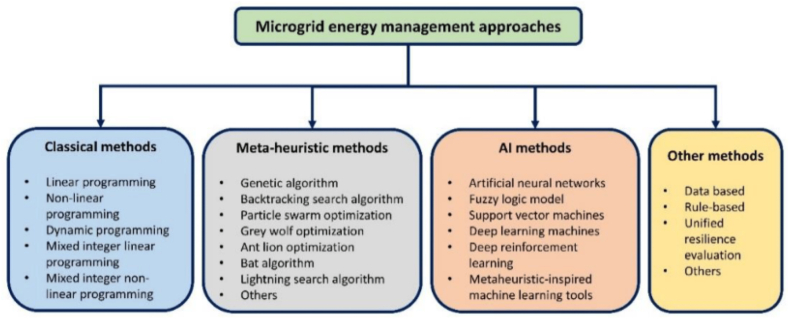
Optimization and management strategies in a microgrid.

The relationship between microgrid constraints and optimization objectives is a fundamental aspect of designing, managing, and operating microgrid systems. Microgrids, as localized and autonomous power networks, are subject to various constraints, encompassing technical, environmental, and economic factors, which can significantly impact their performance. These constraints set boundaries and limitations on what a microgrid can achieve, making it crucial to balance them with the optimization objectives to ensure effective and efficient operation [57]. On one hand, microgrid constraints, such as the availability and variability of renewable energy sources, grid interconnection standards, and environmental impact considerations, serve as the parameters that operators and planners must adhere to. They define the realm within which a microgrid operates and must be carefully managed to prevent grid instability or environmental harm. Additionally, economic constraints, including initial setup costs, ongoing maintenance expenses, and the economic viability of renewable energy sources, affect the financial sustainability of a microgrid [57,58]. On the other hand, optimization objectives encompass the goals and targets that microgrid operators strive to achieve. These objectives often include cost minimization, reduction of carbon emissions, enhanced reliability, and efficient energy management. Optimization strategies seek to make the best use of available resources, adapt to changing conditions, and maximize the benefits of microgrid operation [59]. The interplay between constraints and objectives is where the art and science of microgrid management come into play. Optimization algorithms and control strategies as shown in Fig. 4 bellow are deployed to strike a harmonious balance. For example, load-balancing algorithms ensure that the available energy sources are utilized efficiently while adhering to grid stability constraints. Advanced energy management systems leverage data analytics and artificial intelligence to optimize energy usage while respecting environmental constraints [59,60] (Fig. 4).

In essence, the relationship between microgrid constraints and optimization objectives necessitates a delicate dance. Microgrid operators and planners must navigate these constraints to ensure the system's safe and reliable operation while striving to meet optimization objectives. This involves continuous monitoring, adaptability to changing conditions, and the fine-tuning of control strategies. The ultimate goal is to harness the potential of microgrids as resilient, sustainable, and cost-effective energy solutions while respecting the inherent constraints that guide their operation [61,62].

To achieve those objectives shown in Table 2, we need to modify and adjust some specific parameters of the microgrid that what we will explain in the next chapter [63].

**Table 2:** Some real objectives in energy management.

Objectives	References
Lowering the cost of operation.	[2,48,50,19,22,25,28,32,38,40,41,47,53,[64], [65], [66]]
Lowering the cost of generation	[4,18,20,30,31,34,36,42,67,63,[68], [69], [70], [71]]
Maximizing fuel efficiency and/or battery sizing.	[5,21,23,27,35,59]
Reducing the cost of operating and/or emissions.	[49,29,33,39,44,54,57,72,73]
Reducing the energy used for dumps, emissions, and operation costs	[43]
Reducing both the operating and charging costs (as seen by the client).	[6]
Reducing the power ramping of DGs.	[19]

## 3 Microgrid parameters to adjust for energy management and their impact on the microgrid

**Fig. 5:**
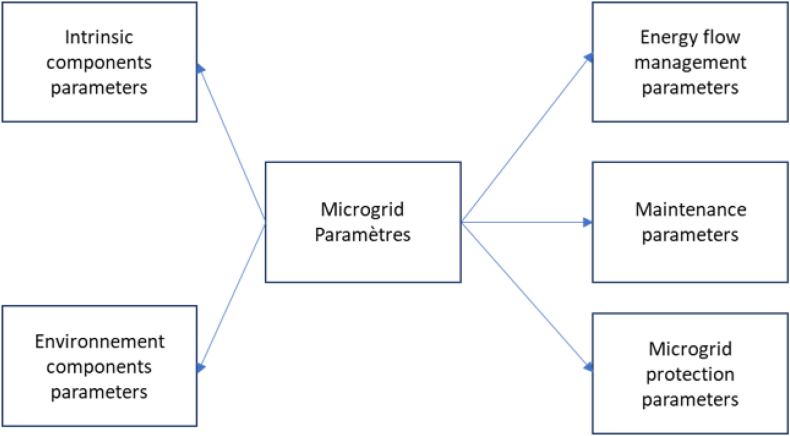
Types of microgrid parameters.

Optimizing energy within a microgrid involves a comprehensive strategy encompassing five key parameter categories [74]. Intrinsic component parameters pertain to the individual components' specifications and capabilities, with the focus on enhancing efficiency and adopting advanced technologies. The interaction between components and the environment considers local conditions, like weather patterns and resource availability, to maximize renewable energy potential. Energy flow management parameters ensure efficient load balancing and minimal transmission losses [74,64]. Maintenance parameters address component upkeep, with preventive measures and real-time monitoring to sustain performance. Microgrid protection parameters focus on safety and protection against operational risks [ [[64], [65], [66]]]. Combining these categories, microgrid operators design a well-rounded optimization approach that emphasizes energy efficiency, cost-effectiveness, environmental goals, and overall reliability while respecting operational constraints [66]. Fig. 5 displays the arrangement of these parameters.

**Fig. 6:**
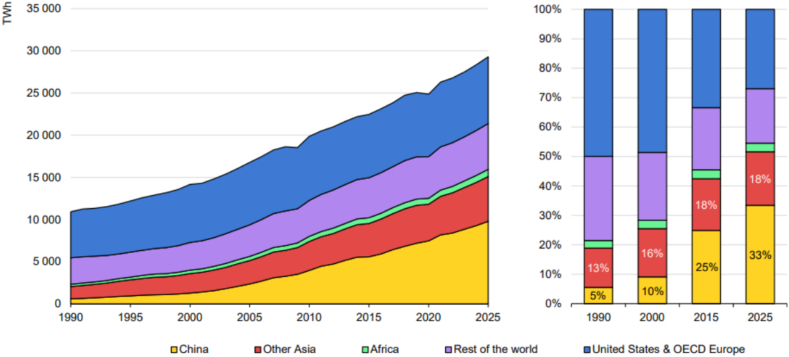
The increasing demand of electricity in the world.

**Fig. 7:**
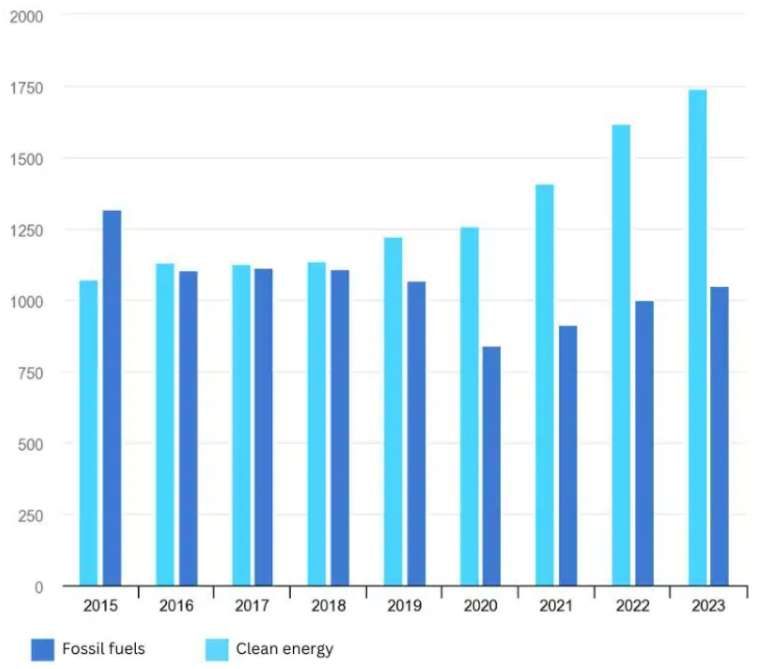
The penetration of renewable energy in the microgrid.

To show the relation and the role various constraint parameters for microgrids, Fig. 6 illustrates the substantial increase in global electricity demand over time, underscoring a significant constraint on microgrid stability and reliability. This escalating demand necessitates a corresponding increase in production capacity to meet the growing needs of consumers while ensuring uninterrupted service delivery. Consequently, microgrid operators are faced with the challenge of enhancing production capabilities to accommodate this rising demand, thereby maintaining grid stability and meeting consumer expectations. In parallel, Fig. 7 highlights the increasing penetration of renewable energy sources into microgrids, emphasizing the need to improve their installation, efficiency, and management. As renewable energy becomes a more prominent component of microgrid generation portfolios, effective integration and optimization of these resources become paramount for achieving sustainability objectives and minimizing reliance on traditional fossil fuel-based generation. Energy Storage Systems (ESS) play a crucial role in facilitating the integration of renewable energy sources by mitigating intermittency and balancing supply-demand dynamics. Thus, optimizing ESS deployment and management becomes a key parameter in microgrid operation, influencing overall system performance and resilience. These figures collectively underscore the multifaceted challenges and opportunities inherent in microgrid management, informing decision-making processes aimed at enhancing grid reliability, sustainability, and cost-effectiveness.

### 3.1 Intrinsic components parameters

The performance of a microgrid depends significantly on the specifications and capabilities of its individual components, such as solar panels, wind turbines, energy storage systems, and backup generators. Optimizing these parameters may include enhancing component efficiency, increasing their capacity, or upgrading to more advanced and efficient technologies [66]. It is important to select sturdy components with an ideal coefficient and cost [68]. As stated in equation (1), the cost considers the component's lifespan as well as its yield, nominal power, purchase, maintenance, and installation costs [68]. The strategic determination of the optimal number of each component within a microgrid represents a pivotal step towards achieving energy optimization. This decision is not just about the selection of components but also the precise calibration of their quantities to harmonize with the energy requirements, thereby striking an ideal balance between supply and demand. Whether it's the number of solar panels, wind turbines, energy storage units, or backup generators, this critical aspect necessitates a comprehensive evaluation of the microgrid's operational needs and environmental conditions. By meticulously aligning the component numbers with the microgrid's specific objectives, one can ensure that the energy generated is neither excessive nor inadequate. This fine-tuned approach not only enhances energy efficiency but also contributes significantly to cost reduction and carbon footprint minimization. The optimization of component numbers, when combined with advanced technologies like AI and IoT, reinforces the microgrid's ability to adapt dynamically to fluctuating energy demands and availability. In essence, this strategic component optimization stands as a key pillar in the quest for a more sustainable, resilient, and cost-effective energy landscape [74,64].

### 3.2 Environment-components parameters

As previously mentioned, wind speed and shading are two random parameters that control how a wind turbine and solar panel operate. however, the two have mediocre yields at 15% for PV and 30% for wind turbine at the edges. A proper PV and wind turbine installation and alignment is required to improve this yield even slightly.

### 3.3 The installation of wind turbine

Wind turbine installation is a complex and highly specialized process that involves several crucial steps. Here's an overview of what's typically involved in installing a wind turbine in Table 3 [65].

**Table 3:** Wind turbine installation parameters.

Wind turbine Installation parameters	Explanation	References
Site Selection and Feasibility Study	Before installing a wind turbine, it's essential to select an appropriate site. This involves evaluating the local wind resources through wind studies and assessing the feasibility of the project. Factors like wind speed, wind consistency, land access, environmental impacts, and grid connection availability are considered.	[46,70]
Permitting and Regulatory Compliance	Obtaining the necessary permits and adhering to local, state, and federal regulations is a critical step. This may involve environmental impact assessments, zoning approvals, and compliance with safety and electrical codes.	[49,42,72]
Foundation Installation	Wind turbines require stable foundations to support their weight and withstand wind forces. There are various foundation types, including concrete slabs, anchor bolts, or monopoles. The choice depends on the turbine size and local conditions.	[49,50]
Transport and Crane Setup	Wind turbine components, including the tower sections, nacelle (housing the generator and gearbox), and rotor blades, are transported to the site. A large crane is used to assemble these components and hoist them into position.	[51]
Tower Erection	The tower sections are assembled, and the tower is erected. It's crucial to ensure that the tower is plumb and level to avoid any structural issues.	[18,19]
Nacelle Installation	The nacelle, which houses the generator and other critical components, is placed on top of the tower. This is a delicate operation that requires precise alignment.	[28]
Rotor Installation	The rotor blades are attached to the hub, creating the turbine's rotor. This step also requires accuracy to ensure proper balance.	[29]
Electrical and Control Systems	Wiring and electrical connections are made to link the turbine to the grid. Control systems are installed to monitor the turbine's performance, respond to changing wind conditions, and ensure safe operation.	[26,28]
Commissioning and Testing	The turbine is thoroughly tested, and its performance is validated. This includes checking the generator, gearbox, and other components, as well as calibrating control systems.	[ 4,18]
Grid Connection	Once the turbine is operational and all tests are successfully completed, it's connected to the electrical grid. This typically involves synchronization and safety measures to ensure a smooth connection	[2,4,75]
Operation and Maintenance	Regular maintenance and monitoring are essential for the turbine's long-term operation. This includes routine inspections, lubrication, and addressing any issues that may arise	[49,51,31]
Safety Measures	Safety is a paramount concern during installation and throughout the turbine's lifecycle. Workers must follow strict safety protocols to prevent accidents	[5,76,70]

Wind turbine installation is a skilled process that requires experienced professionals, precision engineering, and adherence to safety and regulatory guidelines. Proper installation is crucial to ensure the turbine's performance and longevity as a clean and sustainable source of energy.

### 3.4 The orientation of wind turbine

The orientation of a wind turbine, specifically the direction in which it faces, has a significant impact on the turbine's overall performance and power output. To maximize the power generation from a wind turbine, it's crucial to align it correctly with the prevailing wind direction. Here's how the orientation affects output power in Table 4.

**Table 4:** Wind turbine orientation parameters.

Wind turbine orientation parameters	Explanation	References
Aerodynamic Efficiency	Wind turbines are designed with blades that are aerodynamically shaped to capture the kinetic energy of the wind. When a wind turbine faces directly into the oncoming wind (the “upwind" orientation), it experiences the maximum wind speed and flow, allowing the blades to efficiently convert wind energy into mechanical energy. This orientation maximizes the turbine's aerodynamic efficiency, leading to higher power output.	[70,71]
Yaw Control	Wind turbines are equipped with a yaw control mechanism that allows them to align with the wind's direction. This ensures that the turbine is always facing the wind, even as the wind's direction changes. Yaw control is essential for maintaining consistent power output, as it allows the blades to adapt to shifting wind patterns.	[77,78,31]
Avoiding Turbulence	If a wind turbine is not properly oriented into the wind, it can experience turbulence from its own tower or other obstacles, reducing its efficiency. Turbulence disrupts the smooth flow of wind over the blades and leads to power losses. Correct orientation minimizes this issue, enabling the turbine to operate in cleaner, less turbulent air.	[6,10,79]
Optimal Blade Angle	Wind turbines can also adjust the pitch angle of their blades to optimize power output. When properly aligned with the wind, the blades can be set at the ideal angle to capture the maximum energy. Incorrect orientation may require the blades to be pitched at suboptimal angles, leading to decreased efficiency and lower power production.	[70,72,80]
Consistent Output	Proper orientation helps maintain a more consistent power output over time. Wind turbines that face into the wind generate a steadier and more predictable energy supply, which is important for grid integration and the stability of the electrical system.	[79,81]
Energy Yield	The correct orientation ensures that the wind turbine consistently operates near its rated capacity. This results in a higher energy yield, which is essential for maximizing the economic viability of wind energy projects.	[10,79]

In summary, orienting a wind turbine into the wind is critical for harnessing the maximum energy from the wind flow, optimizing aerodynamic efficiency, reducing turbulence, and maintaining consistent power output. Modern wind turbines are equipped with advanced control systems that automatically adjust the orientation to make the most of changing wind conditions, ensuring optimal performance and energy production.

### 3.5 Installation of PV

The installation of photovoltaic (PV) solar panels is a process that involves several steps to ensure the effective and safe setup of a solar energy system. Here's an overview of the typical installation process for PV panels is shown in Table 5.

**Table 5:** PV installation parameters.

PV installation parameters	Explanation	References
Site Assessment and Planning	The first step is to assess the site where the solar panels will be installed. Factors like solar exposure, shading, roof condition, and local regulations are considered Energy needs are also assessed to determine the appropriate size of the PV system.	[3,9]
Design and Permitting	Based on the site assessment, a solar panel system is designed, taking into account the number of panels, their placement, and the inverter capacity. Necessary permits and approvals from local authorities are obtained	[61,62]
Material Procurement	The required solar panels, mounting equipment, inverters, wiring, and other components are procured	[28,74]
Roof Inspection and Reinforcement (If Applicable)	If the solar panels are installed on a roof, the roof's structural integrity may need to be evaluated, and reinforcement may be necessary to support the additional weight	[79,78]
Installation of Mounting Structures	Mounting structures, such as racks or frames, are installed on the roof or the ground. These structures support the solar panels and ensure the correct orientation and tilt angle	[11,82,79]
Wiring and Electrical Work	- Wiring is installed to connect the solar panels to the inverter, which converts the DC (direct current) electricity generated by the panels into AC (alternating current) electricity for use in the building. - A dedicated electrical panel or meter may be added to monitor the solar energy production.	[77]
Solar Panel Installation	Solar panels are securely mounted on the mounting structures. Care is taken to ensure they are positioned correctly to receive maximum sunlight	[44,45]
Inverter Installation	The inverter is installed near the electrical panel or in a convenient location. It's connected to the solar panels and the building's electrical system.	[47,83]
Electrical Connections and Testing	All electrical connections are made, and the system is thoroughly tested to ensure that it functions as expected.	[78,61]
Grid Connection (For Grid-Tied Systems)	If the PV system is grid-tied, it's connected to the utility grid. This may require coordination with the utility company	[3,4,78]
Monitoring and Maintenance	A monitoring system is often set up to keep track of the solar system's performance. Regular maintenance, such as cleaning the panels and checking for any issues, is important to ensure optimal output	[1,3,84]
Final Inspection and Certification	- A final inspection may be carried out to ensure that the installation complies with local codes and safety standards. - After successful inspection, the system may be certified, which may be necessary for receiving incentives or rebates.	[63]

The installation of PV solar panels requires expertise in electrical and structural work. It's often carried out by professional solar installers who are trained to ensure the safe and efficient operation of the system. Proper installation and maintenance are key to the long-term success of a solar energy system, providing clean and renewable energy for many years.

### 3.6 Orientation of PV

The orientation of photovoltaic (PV) solar panels plays a crucial role in maximizing their efficiency and energy production. Proper orientation ensures that the panels receive the maximum amount of sunlight throughout the day. Here are the key aspects to consider in terms of how orientation impacts PV efficiency are shown in Table 6.

**Table 6:** Orientation of PV parameters.

Orientation of PV parameters	Explanation	References
Optimal Angle to the Sun	PV panels are most efficient when they are directly facing the sun, which allows them to capture the maximum amount of sunlight. This alignment is known as the “solar noon position." - For fixed installations, the optimal angle depends on the geographic location of the installation. Generally, in the northern hemisphere, panels are tilted towards the true south, while in the southern hemisphere, they are tilted towards the true north.	[31]
Seasonal Adjustment	- To account for the changing angle of the sun throughout the year, it is often beneficial to adjust the tilt angle of the panels seasonally. - In the winter, the sun is lower in the sky, so tilting the panels up can capture more sunlight. In the summer, the sun is higher, so a flatter angle may be more efficient	[33,34]
East-West Orientation	- PV panels can be oriented either in an east-west direction or north-south. - East-west orientation can be beneficial for maximizing power production throughout the day. Panels facing east capture morning sunlight, while those facing west capture afternoon sunlight.	[[34], [35], [36]]
North-South Orientation	North-south orientation maximizes the annual energy production, as the panels receive a more even distribution of sunlight throughout the day. - This orientation is particularly suitable for grid-tied systems where consistent energy production is essential.	[61,62]
Shading Considerations	- It's crucial to avoid shading on PV panels. Shading, even on a small part of a panel, can significantly reduce the system's overall efficiency. - The orientation should take into account potential shading from nearby trees, buildings, or other obstacles.	[60,61,64]
Tracking system	Tracking systems, like single-axis or dual-axis trackers, can automatically adjust the orientation of PV panels to follow the sun's path throughout the day. These systems can significantly improve energy production, especially in regions with variable weather conditions	[85,86,87]

In the context of optimal solar panel installations, geographical considerations play a crucial role. As highlighted in Table 6, the optimal tilt angle for fixed installations depends on the geographic location of the solar panel system. Specifically, for installations in the Northern Hemisphere, panels are typically tilted towards the true south to maximize sunlight exposure, while in the Southern Hemisphere, the optimal tilt is towards the true north. This adjustment accounts for the sun's path across the sky in different hemispheres, optimizing energy capture throughout the day. By aligning solar panels with these geographic considerations, installations can harness solar energy more efficiently. This principle underscores the importance of tailoring solar panel setups to the specific geographic context, a topic explored in the preceding sections regarding optimal installation practices and factors influencing solar energy generation.

### 3.7 Energy flow management parameters

Energy flow management within a microgrid involves a set of parameters and strategies to ensure efficient and reliable energy distribution. Here are the key energy flow management parameters in a microgrid described in Table 7.

**Table 7:** Energy flow parameters.

Energy flow parameters	Explanation	References
Load Balancing	Load balancing is a critical parameter that involves the even distribution of electrical load among various sources and storage devices within the microgrid. It ensures that the demand for electricity matches the available supply. Effective load balancing prevents overloading of specific components and helps in maximizing the utilization of renewable energy sources.	[11,88,79]
Energy Storage Control	Microgrids often include energy storage systems such as batteries. Managing the state of charge (SoC) and state of health (SoH) of these storage systems is essential for optimizing energy flow. Parameters include setting charge and discharge rates, ensuring efficient energy storage, and controlling the flow of electricity to and from the storage systems.	[10,88,89]
Scheduling and Dispatch	Microgrids may employ scheduling and dispatch strategies to determine when and how energy sources are used. This includes optimizing the use of intermittent renewable resources (e.g., solar and wind) during periods of high availability and minimizing reliance on backup generators during peak renewable generation.	[7,9]
Grid Interaction and Islanding	Microgrids can be connected to the main grid but also designed to operate independently (islanded) during grid disturbances. Parameters related to grid interaction involve the seamless transition between grid-connected and islanded modes to maintain stability and reliability	[61,62]
Demand Response	Demand response programs can be implemented within a microgrid to encourage users to adjust their energy consumption in response to grid conditions. Parameters include setting incentives and communication protocols to facilitate demand response actions.	[32]
Voltage and Frequency Control	Maintaining stable voltage and frequency levels is crucial for reliable operation. Parameters related to voltage and frequency control include adjusting the output of generators, managing energy storage, and implementing load shedding strategies when necessary.	[2,4,6]
Grid Tie-In and Export	Microgrids often have the capability to export excess energy to the main grid. Parameters for grid tie-in involve establishing protocols for energy export, monitoring grid conditions, and controlling the flow of power in both directions.	[70]
Energy Trading and Economic Optimization	In some microgrids, energy trading between different participants (prosumers) can occur. Parameters for economic optimization include setting pricing mechanisms, energy exchange rules, and negotiation strategies	[71,73]
Predictive Analytics and Forecasting	Many microgrids use predictive analytics and forecasting tools to anticipate energy generation, load patterns, and storage requirements. These parameters involve data-driven models and algorithms that help optimize energy flow management.	[69,70]
Microgrid Control Systems	Centralized or distributed control systems manage the parameters mentioned above. The parameters within these control systems include logic and algorithms that monitor, analyze, and adjust the energy flow in real-time	[90,91]

Effective energy flow management parameters are designed to ensure energy availability, reliability, and efficiency within a microgrid, while also maximizing the utilization of renewable energy sources and minimizing operational costs. These parameters may vary depending on the specific objectives and design of the microgrid, as well as local conditions and regulations.

Fine-tuning energy flow parameters allows us to precisely match electricity production with dynamic load requirements, preventing unnecessary overproduction. Taking the example of a household with a maximum load of 12 kW, an average load of 5 kW, and a minimum load of 2 kW, effective management ensures that the system responds dynamically to the load demand. By avoiding excess production, we not only optimize energy usage in real-time but also adhere to the average load requirement without exceeding it. This strategic energy management not only aligns with environmental sustainability goals by reducing Carbon Dioxide emissions but also translates into cost savings. Given that the production of energy involves Carbon Dioxide emissions and comes with a price, efficient energy management results in lower costs and minimized environmental impact.

### 3.8 Maintenance parameters

Maintaining a microgrid is essential for ensuring its long-term reliability and optimal performance. Maintenance parameters encompass a range of activities and considerations that help keep the microgrid components in good working condition. Here are the key maintenance parameters for a microgrid shown in Table 8.

**Table 8:** Maintenance parameters.

Maintenance parameters	Explanation	References
Scheduled Inspections	Regular inspections of all microgrid components, including generators, solar panels, wind turbines, energy storage systems, and control equipment, are essential. Scheduled inspections help identify potential issues before they lead to system failures	[69]
Preventive Maintenance	Maintenance schedules should include preventive tasks such as cleaning, lubrication, and tightening of connections. These tasks prevent wear and tear and prolong the life of components.	[70,71]
Testing and Calibration	Regular testing and calibration of equipment, including sensors, meters, and control systems, are crucial for ensuring accurate measurements and optimal system performance	[67,83]
Battery Health Monitoring	If the microgrid includes energy storage systems, monitoring battery health is critical. Regular checks on the state of charge (SoC), state of health (SoH), and overall battery performance help prevent unexpected failures.	[54,55,57]
Generator Maintenance	For microgrids with backup generators, maintenance includes checking fuel levels, oil changes, and regular testing to ensure generators are ready to operate during power outages	[3,82]
Control System Updates	Control software and hardware should be regularly updated to include bug fixes, security patches, and performance enhancements. This ensures the stability and security of the microgrid control system.	[1,5,79]
Data Management and Analysis	Regular data analysis helps in identifying performance trends and anomalies. Advanced analytics can predict component failures and optimize maintenance schedules	[11,79]
Equipment Replacement	Components with a limited lifespan, such as batteries or certain electrical equipment, may need to be replaced at the end of their service life to maintain system reliability	[3,5,7]
Environmental Considerations	Environmental factors, such as extreme weather conditions or exposure to corrosive elements, may require additional maintenance. Protective measures, like weatherproofing or corrosion-resistant coatings, should be considered.	[8,7,10]
Safety Inspections	Safety inspections are critical to ensure that the microgrid complies with safety standards and regulations. This includes checking for electrical hazards, fire risks, and other safety concerns.	[2,8,10]
Emergency Response Planning	Having a well-defined emergency response plan is essential. This plan should outline procedures for addressing unexpected failures, outages, and other emergencies, ensuring a swift and coordinated response.	[5,8,79]
Documentation and Records	Keeping detailed maintenance records, including maintenance logs, inspection reports, and equipment manuals, is essential for tracking the maintenance history of each component and facilitating audits or warranty claims	[2,4,7]
Personnel Training	Maintenance personnel should receive ongoing training to stay updated on best practices, safety procedures, and the latest technologies in the microgrid industry.	[3,75,78]
Remote Monitoring	Many microgrids incorporate remote monitoring and control systems that allow for real-time monitoring of performance. These systems can help identify issues and initiate remote troubleshooting or maintenance actions.	[82,79,78]

The maintenance parameters for a microgrid are designed to maximize the system's uptime, extend the lifespan of its components, and ensure its continued reliability. Routine and proactive maintenance is crucial for achieving these objectives and for the long-term success of the microgrid.

### 3.9 Microgrid protection parameters

Safety is of utmost importance in microgrid operations to protect people, property, and the environment. Microgrid safety parameters encompass a wide range of considerations and protocols to ensure secure and reliable operation. Here are the key safety parameters for a microgrid described in Table 9.

**Table 9:** Microgrid protection parameters.

Microgrid protection parameters	Explanation	References
Electrical Safety	- Ensuring that all electrical equipment, wiring, and connections meet safety standards and codes. - Proper grounding and bonding of equipment to prevent electrical hazards. - Regular electrical inspections and maintenance to prevent short circuits and electrical fires.	[60,63]
Overcurrent Protection	- Installing overcurrent protection devices (fuses, circuit breakers) to safeguard against excessive current and prevent damage to equipment. - Proper sizing and coordination of protection devices to prevent circuit overloads.	[5,18]
Fire Safety	- Fire prevention measures, such as the use of fire-resistant materials in construction and equipment. - Installation of fire detection and suppression systems. - Fire safety training for personnel operating within or near the microgrid.	[79,77]
Emergency Shutdown and Isolation	- Implementing emergency shutdown procedures and safety switches to isolate electrical equipment during maintenance or emergencies. - Lockout/tagout (LOTO) procedures to ensure that equipment is de-energized and safe for maintenance.	[92,93]
Equipment Safety Standards	- Ensuring that all microgrid components and equipment comply with industry safety standards and certifications. - Regular equipment inspections and testing to verify safety compliance.	[94,90]
Hazardous Materials Handling	- Proper handling and storage of hazardous materials, such as batteries, fuel, and lubricants, to prevent leaks or spills. - Establishing protocols for responding to hazardous material incidents	[73,80]
Personnel Training	- Training for all personnel on microgrid safety procedures, including emergency response, evacuation, and first aid. - Specific training for individuals operating or maintaining microgrid equipment.	[95]
Electromagnetic Compatibility (EMC)	- Ensuring that electromagnetic interference (EMI) and electromagnetic compatibility (EMC) are managed to prevent interference with sensitive electronic equipment.	[69]
Environmental Protection	- Implementing measures to protect the environment, including spill containment and waste disposal practices. - Compliance with environmental regulations to prevent pollution.	[70,95]
Lightning Protection	- Installing lightning protection systems to safeguard against lightning strikes, which can damage equipment and pose safety risks.	[94]
Public Safety and Accessibility	- Ensuring that microgrid facilities are secure and that public access is restricted to protect against accidents and unauthorized access.	[90,91]
Safety Protocols for Grid Disconnection and Reconnection	- Safe procedures for disconnecting the microgrid from the main grid and for reconnection after outages. - Safety measures to prevent back-feeding of power into the grid during outages.	[[90], [94], [95]]
Emergency Power Sources	- Backup power sources, such as emergency generators or uninterruptible power supplies (UPS), to provide power during grid outages and critical operations.	[40]
Remote Monitoring and Control	- Remote monitoring systems that allow for real-time safety and performance monitoring, as well as remote control to respond to safety issues promptly.	[71]
Emergency Communication Systems	- Reliable communication systems to coordinate responses during emergencies and maintain contact with emergency services.	[70,71]

Microgrid safety parameters are critical for protecting the safety of personnel and the community, as well as ensuring the resilience and reliability of the microgrid. These parameters should be integrated into the design, operation, and maintenance of the microgrid to minimize risks and prevent accidents.

## 4 Discussion of the relevance of our findings in the light of current trends and future perspectives in microgrid technology

The review, titled “Constraints and Adjustable Parameters in Microgrids for Cost and CO2 Emission Reduction," is strategically positioned within the current landscape of microgrid technology, where sustainability and efficiency are paramount. In the context of prevailing trends, our findings bear significant relevance. With a heightened emphasis on sustainable energy practices, the identification and comprehensive analysis of constraints within microgrids, coupled with the exploration of adjustable parameters for cost and CO2 emission reduction, are timely and essential. The global shift towards renewable energy integration and the rapid advancement of energy storage technologies underscore the pertinence of our study. As microgrids evolve into pivotal components of future energy systems, our review offers crucial insights into the factors influencing their performance. In particular, our exploration of adjustable parameters, such as load balancing strategies, demand response mechanisms, and energy storage optimization, aligns seamlessly with the contemporary drive towards decentralized and resilient energy solutions. The adaptability of microgrids to dynamic energy demands, as highlighted in our review, positions them as integral players in the ongoing transition to more sustainable and environmentally friendly energy practices.

Looking forward, our review serves not only as a snapshot of the current state of microgrid technology but also as a guiding document for future research and development endeavors. The adjustable parameters elucidated in our study provide a concrete pathway for future investigations, offering a blueprint for refining and optimizing microgrid designs. By addressing the challenges and harnessing the potential identified in our review, the microgrid community can collectively contribute to achieving enhanced cost-effectiveness and a substantial reduction in carbon emissions. In this way, our work not only encapsulates the present concerns of microgrid technology but also lays the groundwork for its promising future. The future perspectives for microgrids are intricately tied to emerging technologies such as the Internet of Things (IoT), Artificial Intelligence (AI), and blockchain. Here's an elaboration on how these technologies could shape the future of microgrid development.

### 4.1 Integration with IoT

The seamless integration of microgrids with IoT technologies holds immense promise for enhancing their functionality. By deploying a network of sensors and smart devices throughout the microgrid infrastructure, real-time data on energy consumption, generation, and equipment performance can be gathered. This wealth of data enables dynamic monitoring and control, facilitating more precise load balancing, predictive maintenance, and the optimization of energy resources. Moreover, IoT connectivity can empower end-users with insights into their energy usage, fostering greater awareness and encouraging energy-efficient behaviors.

### 4.2 AI-driven optimization

Artificial Intelligence, particularly machine learning algorithms, can revolutionize the way microgrids operate. AI-driven optimization can analyze vast datasets generated by IoT devices to identify patterns, forecast energy demand, and adapt the microgrid's parameters in real-time. Machine learning algorithms can optimize energy storage, predict equipment failures, and even automate decision-making processes. This capability is crucial for ensuring the efficient utilization of resources, cost reduction, and the minimization of carbon emissions, aligning perfectly with the goals of your review on cost and Carbon Dioxide emission reduction in microgrids.

### 4.3 Blockchain for transparent and secure transactions

Blockchain technology offers a decentralized and secure approach to managing transactions and data in microgrids. Through the use of smart contracts, blockchain ensures transparent and verifiable energy transactions within the microgrid ecosystem. This can enable peer-to-peer energy trading, where users can directly buy and sell excess energy, fostering a more decentralized and resilient energy market. Additionally, the transparent and tamper-proof nature of blockchain enhances the security of critical energy data and transactions, addressing concerns related to cybersecurity and data integrity.

### 4.4 Decentralized energy management with distributed ledger

Building on the principles of decentralization, microgrids can leverage distributed ledger technologies to establish a consensus-driven approach to energy management. A distributed ledger could be used to record and validate transactions, ensuring that energy transactions are secure, transparent, and traceable. This decentralized approach aligns with the inherent structure of microgrids, where localized generation and consumption are prevalent.

In summary, the future of microgrids is intrinsically tied to the transformative potential of IoT, AI, and blockchain technologies. The integration of these technologies can usher in an era of intelligent, adaptive, and decentralized energy systems, aligning with the aspirations outlined in your review on constraints and adjustable parameters for microgrid cost and Carbon Dioxide emission reduction.

## 5 Conclusion

In conclusion, the transformative potential of microgrids in reshaping the energy landscape is evident, with clear benefits in enhancing efficiency, reducing costs, and mitigating carbon emissions. Microgrids, adept at navigating diverse constraints, both inherent and environmental, are crucial in ushering in a sustainable and eco-conscious energy era. This necessitates a nuanced understanding and adept management of technical limitations, as well as external variables such as weather patterns and grid dynamics. The journey towards optimal microgrid performance lies not only in overcoming these challenges but also in capitalizing on the revolutionary synergy between emerging technologies. Artificial Intelligence (AI) and the Internet of Things (IoT) emerge as pivotal game-changers in this context. The data-driven decision-making capabilities of AI and the interconnected nature of IoT devices present a myriad of possibilities for microgrid management. In particular, AI algorithms, leveraging real-time data, can accurately predict energy demand, optimize the utilization of renewable resources, and proactively identify potential component failures. Simultaneously, IoT devices embedded in various components provide valuable insights into energy flow and load balancing. By harnessing the power of AI and IoT, microgrids can make precision adjustments to energy management parameters in real time, effectively achieving the dual objectives of cost reduction and Carbon Dioxide emission minimization. These technologies empower microgrids to swiftly adapt to changing conditions, maximize the use of renewable energy sources, and decrease reliance on fossil fuels. Additionally, they open avenues for active participation in demand response programs, contributing significantly to grid stability and related services. In essence, the harmonious integration of microgrid constraints, energy management parameters, and AI-powered IoT systems delineates a clear pathway to a cleaner, more sustainable, and cost-effective energy future. The dynamic optimization and adaptability offered by these technologies not only meet current energy needs but also pave the way for a greener and more efficient energy landscape. Microgrids, in this narrative, assume a central role in not just reducing costs and Carbon Dioxide emissions but actively promoting environmental sustainability. In reflection of the main findings of this study, our exploration into the transformative potential of microgrids reveals their significant impact on reshaping the energy landscape. The comprehensive integration of AI and IoT technologies not only addresses the challenges associated with microgrid management but propels these systems to new heights of efficiency and sustainability. The utilization of AI algorithms for real-time prediction of energy demand, coupled with the insights provided by IoT devices, has showcased the remarkable adaptability of microgrids to changing conditions. The precision adjustments enabled by these technologies have resulted in a dual achievement – substantial cost reduction and a noteworthy decrease in CO2 emissions. Our research underscores that microgrids, empowered by AI and IoT, play a central role in steering us towards a cleaner, more sustainable, and economically viable energy future.

## Funding

The research is partially funded by the 10.13039/501100012190Ministry of Science and Higher Education of the Russian Federation as part of the World-class Research Center program: Advanced Digital Technologies (contract No. 075-15-2022-312 dated April 20, 2022).

## Data availability statement

Data will be made available on request.

## CRediT authorship contribution statement

**Mohammed Amine Hoummadi:** Writing – original draft, Methodology, Investigation, Formal analysis, Data curation, Conceptualization. **Hala Alami Aroussi:** Software, Resources, Methodology, Investigation. **Badre Bossoufi:** Validation, Supervision. **Mohammed Karim:** Validation, Resources, Project administration. **Saleh Mobayen:** Methodology, Investigation, Funding acquisition, Formal analysis. **Anton Zhilenkov:** Visualization, Resources, Methodology, Funding acquisition. **Thamer A. H. Alghamdi:** Writing – review & editing, Software.

## Declaration of competing interest

The authors declare that they have no known competing financial interests or personal relationships that could have appeared to influence the work reported in this paper.
